# Perception and practice of Kangaroo Mother Care after discharge from hospital in Kumasi, Ghana: A longitudinal study

**DOI:** 10.1186/1471-2393-11-99

**Published:** 2011-12-01

**Authors:** Samuel B Nguah, Priscilla NL Wobil, Regina Obeng, Ayi Yakubu, Kate J Kerber, Joy E Lawn, Gyikua Plange-Rhule

**Affiliations:** 1 Department of Child Health, Komfo Anokye Teaching Hospital, P. O. Box 1934, Kumasi, Ghana; 2 Suntreso Government Hospital, Private mail bag, Kumasi, Ghana; 3 Saving Newborn Lives/Save the Children USA, 11 South Way, Cape Town 7405, South Africa; 4 Department of Child Health, School of Medical Sciences, Kwame Nkrumah University of Science and Technology, Kumasi, Ghana

## Abstract

**Background:**

The practice of Kangaroo Mother Care (KMC) is life saving in babies weighing less than 2000 g. Little is known about mothers' continued unsupervised practice after discharge from hospitals. This study aimed to evaluate its in-hospital and continued practice in the community among mothers of low birth weight (LBW) infants discharged from two hospitals in Kumasi, Ghana.

**Methods:**

A longitudinal study of 202 mothers and their inpatient LBW neonates was conducted from November 2009 to May 2010. Mothers were interviewed at recruitment to ascertain their knowledge of KMC, and then oriented on its practice. After discharge, the mothers reported at weekly intervals for four follow up visits where data about their perceptions, attitudes and practices of KMC were recorded. A repeated measure logistic regression analysis was done to assess variability in the binary responses at the various reviews visits.

**Results:**

At recruitment 23 (11.4%, 95%CI: 7.4 to 16.6%) mothers knew about KMC. At discharge 95.5% were willing to continue KMC at home with 93.1% willing to practice at night. 95.5% thought KMC was beneficial to them and 96.0% beneficial to their babies. 98.0% would recommend KMC to other mothers with 71.8% willing to practice KMC outdoors.

At first follow up visit 99.5% (181) were still practicing either intermittent or continuous KMC. This proportion did not change significantly over the four weeks (OR: 1.4, 95%CI: 0.6 to 3.3, p-value: 0.333). Over the four weeks, increasingly more mothers practiced KMC at night (OR: 1.7, 95%CI: 1.2 to 2.6, p = 0.005), outside their homes (OR: 2.4, 95%CI: 1.7 to 3.3, p < 0.001) and received spousal help (OR: 1.6, 95%CI: 1.1 to 2.4, p = 0.007). Household chores and potentially negative community perceptions of KMC did not affect its practice with odds of 0.8 (95%CI: 0.5 to 1.2, p = 0.282) and 1.0 (95%CI: 0.6 to 1.7, p = 0.934) respectively. During the follow-up period the neonates gained 23.7 sg (95%CI: 22.6 g to 24.7 g) per day.

**Conclusion:**

Maternal knowledge of KMC was low at outset. Once initiated mothers continued practicing KMC in hospital and at home with their infants gaining optimal weight. Continued KMC practice was not affected by perceived community attitudes.

## Background

In sub-Saharan Africa, 14 percent of babies are born LBW, a birth weight of less than 2500 g. Most newborn babies who die are LBW accounting for 60 to 90 percent of newborn deaths globally [1]. There is little evidence for prevention of preterm births and LBW even in high-resource settings. A review of 2000 studies by Barros et al and the Global Alliance for the Prevention of Prematurity and Stillbirth (GAPPS) Review Group in 2010 [2] showed a paucity of data on the impact of potentially relevant interventions for the prevention and management of preterm births for low and middle income countries like Ghana [2]. In this global report, 11 interventions including hospital-based Kangaroo Mother Care (KMC), were found to improve the survival of preterm or LBW babies. Identifying small babies and providing extra support for feeding also has great potential to reduce neonatal mortality rate [1]. KMC is an intervention for caring and improving the outcome of preterm babies. KMC started in 1978 in Colombia as a way of dealing with problems of separation of mother and baby, overcrowding, and scarcity of incubators in hospitals caring for low birth weight infants [3]. KMC refers to skin-to-skin contact between mother and baby thereby providing warmth, promoting exclusive breastfeeding and facilitating early discharge from hospital. It has been proposed as an alternative to conventional or incubator care for LBW infants [4]. Other caregivers can provide KMC with other members of the family, including the babies' fathers and grandmothers, without disrupting breastfeeding routines. KMC usually starts in hospital, is continued at home after discharge with routine follow up visits scheduled to weigh the baby, counsel on feeding and check for danger signs [5].

Many studies have reported the benefits of KMC over incubator care [6,7]. Evidence backs the effectiveness and safety of KMC in stable, preterm infants. In LBW infants weighing 2000 g or less, who are unable to regulate their temperature, KMC is at least as safe and effective as incubator care [5,8]. An open randomised controlled trial in Bogotá, Colombia [9], assessing the long term clinical effects of KMC found that KMC improved successful breastfeeding rates and infections were milder in these children. A Cochrane review [4] in 2003 did not find any mortality benefit with KMC but a recent systematic review by Lawn et al [10] 2010 showed that KMC substantially reduces neonatal mortality and morbidity especially due to infections, among preterm babies in hospital with a weight of ≤2000 g.

Few studies have documented the effectiveness of KMC once the mothers are discharged from hospital into their various communities in which they live in low income settings. A randomised controlled trial in rural Bangladesh looking at home initiation of KMC for all babies showed that KMC practice was more common among mothers who were taught and counselled on KMC compared to those who were not, but no mortality effect was reported [11]. Another study also in Bangladesh reported that KMC practice in the community was quickly and popularly adopted by mothers after delivery [12]. To date no study has documented the continued practice of KMC amongst mothers and babies once they return to their communities without active follow up.

KMC was first introduced in Ghana in 2007 and currently being practiced in six out of ten regions. Its practice usually starts in hospital with mothers being encouraged to continue at home. This continuation however depends heavily on local circumstances in the home and community. Though there appears to be success in initiating parents into the practice of KMC at facilities, very little evidence exist to support the continued practice of KMC and follow up at peripheral health facilities once the mother and baby are discharged. This study was therefore designed to evaluate the practice of KMC among mothers who are initiated in the hospital and sent home to continue practicing KMC without active supervision by health personnel or strict adherence to a study protocol.

## Methods

### Study design

This was a longitudinal study of mothers with LBW newborns who were willing to practice KMC at the Mother Baby Units of the Komfo Anokye Teaching Hospital (KATH) and the Suntreso Government Hospital (SGH) in Kumasi, Ghana.

### Study sites

Kumasi is the capital of the most populous region in the country, the Ashanti region. With a population just under two million, Kumasi is the second largest city in Ghana and a typical African urban city. Families often live with extended family members who influence many of the decisions made in the household [13]. It is traditional among many Ghanaians even in urban areas to either move in to live with their mothers or have their mothers to move in around the time of childbirth. These grandmothers are highly influential in making decisions concerning the care of the newborns [14].

The study was conducted in two hospitals in Kumasi-KATH and SGH. KATH is a teaching hospital that provides tertiary care, while SGH is a district hospital and provides secondary care. There are three Mother-Baby Units in Kumasi and these units are at KATH, SGH and the Kumasi South Hospital (Regional Hospital). Collaboration between KATH and SGH has enabled moderately sick newborns not requiring specialised care to be transferred to the latter to continue with their treatment. SGH is about 5 km from KATH while KSH is about 15 km away from KATH. Because of the proximity of SGH to KATH, it was selected as one of the study sites.

The KATH Mother-Baby Unit runs a 24 hour in-patient service and admits about 4000 infants less than three months old every year, with about a quarter of these being preterm. It receives referrals from district and private hospitals, maternity homes as well as home deliveries from the Kumasi metropolis and the northern sector of the country. The Unit has 77 cots, 4 functioning incubators, 2 overhead radiant heaters, 10 phototherapy units and 20 oxygen delivery outlets. The unit is overcrowded with 3 or 4 babies sharing one radiant heater or incubator, and at times 2 or 3 babies sharing a cot. KMC has therefore become a vital and indispensable intervention for preterm and LBW babies in the unit. Continuous KMC (placing the infant in skin to skin contact round the clock) would have been ideal but the KATH Mother-Baby Unit lacks space. The unit has three main wings-a High Dependency Unit, a preterm and intermittent KMC unit, and a Septic unit. Preterm babies are admitted for special care and intermittent KMC (placing the infant in skin to skin contact for less than 24 hours). Due to limited space in the overcrowded unit, mothers are accommodated in a separate room and are only able to practice intermittent KMC for approximately four to six hours in the day, mainly after breastfeeding.

The SGH Mother-Baby Unit admits neonates transferred from KATH or referred from other district hospitals and facilities. The unit has three wards-one postnatal ward for sick neonates to room-in with their mothers, one neonatal care ward for babies in need of resuscitation, oxygen and phototherapy, and a KMC ward. The postnatal ward has 16 beds and KMC is encouraged to take place there as well. Continuous KMC is practiced at SGH as there is enough space for rooming in. The neonatal care ward has 13 beds, and the KMC ward has 4 beds. The Mother-Baby Unit runs a 24 hour service and is staffed by a paediatrician and seven nurses. As part of routine care before discharge from both Mother-Baby Units, mothers are encouraged to continue practicing KMC at home and bring their babies for weekly follow up care till they attain a weight of 3000 g.

### Sample size

To detect an overall two sided change in the proportion of mothers practicing KMC by 10%, the proportion of mothers practicing KMC at first visit was assumed to be 80%. The sample size required for a four repeated binary outcome with 80% statistical power and a two-sided 0.05 significance level in a balanced longitudinal design was calculated to be 157. Assuming a 25% dropout or loss to follow-up rate, a final sample size of 196 was required for the study if an interclass correlation among measurements within subjects is assumed to be 0.5.

### Study population

Neonates admitted to either the KATH Mother-Baby Unit or the SGH Mother-Baby Unit from November 2009 through May 2010 were eligible for the study. However, only those weighing between 1000 g and 2000 g and less than a week old, whose mothers were willing to practice KMC, lived within a distance of ten kilometres from the hospital and gave consent, were included in the study. Very sick neonates with life threatening conditions, major congenital abnormalities or requiring intensive care were excluded.

### Study Procedure

All neonates brought to the KATH Mother-Baby Unit and SGH Mother-Baby Unit were first admitted and stabilized. For those who fulfilled all the inclusion criteria and none of the exclusion criteria, the study was fully explained to the mother and or legal guardian. The mothers and or guardians were then taken through a brief but comprehensive training session on the practice of KMC by an experienced nurse who has been trained in teaching KMC. A verbal consent was then obtained from the mothers or legal guardians and neonates were recruited into the study at this point if their mothers or guardians were still willing to participate in the study. Verbal consent was sought because practice of KMC was routine for mothers of low birth weight babies on admission.

After recruitment, clinical data was collected using a pre-tested questionnaire and included variables such as the birth weight, mode of delivery, gestational age, type of KMC and weight of infant at initiation of KMC. Mothers were supported by a nurse during their first attempts at practicing KMC to ensure the technique was right before they continued to practice without supervision. Mothers also helped each other in initiating the KMC position. Neonates admitted to both Mother-Baby Units were monitored until discharge or death.

Upon discharge from either Mother-Baby Unit data was collected on the infant, including discharge weight, duration of stay in hospital and mode of feeding. The mothers were then interviewed to ascertain their perception of the usefulness, challenges and benefits of practicing KMC to themselves and their babies. They were also asked whether or not they thought their spouses would help practice KMC when they got home. Finally they were asked about how they envision the community's acceptance of the practice of KMC both within and outside their homes. They were then encouraged to practice continuous KMC at home and to come for weekly reviews for the next four weeks. Only the mothers' telephone numbers were taken at recruitment. They were assured no Mother-Baby Unit or study staff would be visiting them at home or would monitor their practice of KMC at home. This was to assure them their continued practice at home will be purely voluntary with no compulsion whatsoever.

After discharge the mothers or guardians together with their babies came for follow up visits weekly for four consecutive visits. The infants' weight, length, head circumference, type of KMC practiced since last seen, and feeding practices were recorded at every visit. The mother or guardian was also asked about any challenges they faced practicing KMC since their last review or discharge. The data collected included perception of the benefits and challenges of KMC to them and their babies. Also, data were recorded on the community and spousal acceptability, support or challenges mothers encountered in their practice of KMC since their last visit or discharge from hospital. Mothers who missed their review appointment were called on telephone several times and reminded. However, those who could not be reached on phone or still did not come for their appointment were not traced to their homes.

### Statistical analysis

Completed case report forms were double entered using Microsoft Access^® ^2007, compared and cleaned for anomalous data. The clean data was then transferred to Stata/SE version 11.1 for analysis. Continuous variable such as the birth weight and gestational ages were summarised and presented as means with their 95% confidence intervals and ranges. Categorical variables were analysed and presented as proportions with their binomial exact 95% confidence intervals. Comparison between the birth and discharge weights was done using a paired t test. A repeated measure logistic regression analysis was done for the binary responses at the various reviews. Cumulative changes in the proportions of these binary outcomes over the four review visits were expressed as odds ratios with their 95% confidence intervals. These odds were then adjusted for the weight at review as it was reasonable to assume KMC practice will decrease as the infants gain weight and grew older. In reporting, a two sided p-value less than or equal to 0.05 was considered statistically significant.

### Ethical considerations

The Committee on Human Research, Publications and Ethics of Kwame Nkrumah University of Science and Technology, Kumasi gave ethical clearance for this study after reviewing the study protocol. All mothers or legal guardians provided verbal informed consent before any study related information was obtained. The mothers or guardians were, however, assured of strict anonymity in answering the questions.

## Results

**Figure 1:**
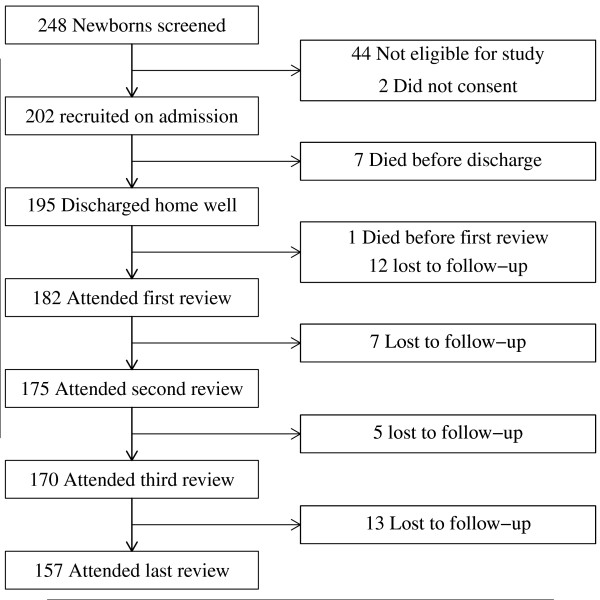
**Flow of eligibility, recruitment and reviews among study participants**.

Two hundred and two newborns out of the 248 screened were recruited from November 2009 through May 2010. 31 were ineligible because they were too ill, mainly with signs of respiratory distress, 11 because they lived more than 10 kilometres away from the hospital, 2 because they were not willing to practice KMC and 2 because they did not provide consent. Mothers for all recruited infants were available to practice KMC. A flow chart of participants in the course of the study is as shown in Figure 1. In all 81.0% (157/195) of the recruited infants were successfully followed up for the entire study follow up period.

All neonates admitted at KATH Mother-Baby Unit (165) initially practiced intermittent KMC with those admitted at SGH Mother-Baby Unit (37) practicing continuous KMC whiles on admission (Table 1). The mean birth weight and gestational age of the neonates were 1600 g (SD: 0.3, Range: 800 g to 2200 g) and 31 weeks (SD: 3.1, Range: 28 to 38 weeks) respectively (Table 2). One hundred and seventy one mothers (84.6%) initiated KMC within 24 hours of birth, 16 (7.9%) initiated KMC after 24 hours but before 48 hours of birth with the rest, 15 (7.4%) initiating after 48 hours but within a week of delivery. Seven neonates (3.5%, 95%CI: 0.9 to 06.0%) died before discharge from the Mother-Baby Unit. At recruitment only 23 (11.4%, 95%CI: 7.4 to 16.6%) mothers knew about KMC. The median duration of admission at both Mother-Baby Units was 12 days (IQR: 8 to 17 days).

**Table 1:** Type of KMC practiced by mothers at various visits

	KMC type practiced n(%)		
	
Stage of study (n)	Not practicing	Intermittent	Continuous
Mother Baby Unit (n = 202)	0 (0.0)	165 (81.7)	37 (18.3)
First review visit (n = 182)	1 (0.5)	65 (37.5)	116 (63.7)
Second review visit (n = 175)	3 (1.7)	42 (24.0)	130 (74.3)
Third review visit (n = 170)	5 (2.9)	45 (24.1)	124 (72.9)
Fourth review visit (n = 157)	9 (5.7)	38 (24.2)	110 (70.1)

**Table 2:** Clinical data at various periods on admission

	Period		
	
	At Birth (n = 202)	Initiation of KMC (n = 199)	At Discharge (n = 195)
Weight (grams) Mean (SD)	1591 (296)	1530 (286)	1573 (294)
Estimated Gestational age (weeks) Mean(SD)	31.5 (3.1)	_	_
Death n(%)	_	3 (1.5%)	7 (3.5%)
Admission Duration (Days) Median(IQR)	_	_	12 (8-17)
Caesarean section delivery n(%)	45 (22.3%)	_	_
Baby taking top-up Expressed Breast Milk n(%)	_	_	180 (89.1%)
Baby actively suckling n(%)	_	_	188 (93.1%)
Baby on exclusive breastfeeding n(%)	_	_	190 (94.1%)

At discharge 188 (93.1%, 95%CI: 88.6 to 96.2%) were actively suckling, 190 (94.1%, 95%CI: 89.8 to 96.9%) were exclusively breastfeeding and 180 (89.1%, 95%CI: 84.0 to 93.0%) were receiving top up expressed breast milk (additional expressed breast milk given to the infant after baby has finished actively suckling when put to breast). Most of the mothers had favourable opinion of the practice of KMC. 95.5% had decided they were going to continue KMC at home, 93.1% were willing to practice KMC at night, 95.5% thought KMC was beneficial to them, 96.0% said KMC was beneficial to their babies and 98.0% were willing to recommend KMC to other mothers (Table 3). However, lesser proportions were willing to practice KMC outside their homes (71.8%) and thought KMC was easy to practice (61.9%).

**Table 3:** KMC perception, intended practice and knowledge of mothers at discharge

		Number	Proportion	
		(n = 202)	(%)	95% CI
Mother will continue practicing KMC at home?	Yes	193	95.5	91.7-97.9
Mother will practice KMC at night?	Yes	188	93.1	88.6-96.2
Mother will practice KMC outside her house?	Yes	145	71.8	65.0-77.9
Cord not fallen off would not prevent KMC?	Yes	177	87.6	82.3-91.8
KMC beneficial to mother?	No	5	2.5	0.8-5.7
	Yes	193	95.5	91.7-97.9
	Not sure	4	2.0	0.5-5.0
KMC beneficial to baby?	No	4	2.0	0.5-5.0
	Yes	194	96.0	92.3-98.3
	Not sure	4	2.0	0.5-5.0
Easy practicing KMC?	No	75	37.0	30.4-44.2
	Yes	125	61.9	54.8-68.6
	Not sure	2	1.0	0.1-3.5
Spousal's knowledge of KMC?	No	34	16.8	12.0-22.7
	Yes	166	82.2	76.2-87.2
	Not sure	2	1.0	0.1-3.5
Husband/helper to help practice KMC?	No	44	21.8	16.3-28.1
	Yes	155	76.7	70.3-82.4
	Not sure	3	1.5	0.3-4.3
Husband to allow KMC practice at night?	No	8	4.0	1.7-7.6
	Yes	176	87.1	81.7-91.4
	Not sure	18	8.9	5.4-13.7
Mother to recommend KMC to others?	No	2	1.0	0.1-3.5
	Yes	198	98.0	95.0-99.5
	Not sure	2	1.0	0.1-3.5
Percieved community attitude towards KMC?	Surprise	135	66.8	59.9-73.3
	Make Fun of	8	4.0	1.7-7.6
	To know more	19	9.4	5.8-14.3
	Supportive	4	2.0	0.5-5.0
	No Idea	36	17.8	12.8-23.8

At discharge 82.2% of the mothers indicated their spouses were aware of KMC, 87.1% were confident their spouses would allow them to practice KMC at night but a lesser number, 76.7%, thought their spouses or helpers at home would actually help practice KMC. Only 2.0% thought members of the community will be supportive.

At the first post discharge review, 99.5% of the mothers were practicing KMC at home with 63.7% practicing continuous KMC (Table 4). The numbers practicing KMC at home seemed to reduce significantly with each visit but after adjusting for the infants' weight, it was observed that the proportions remained relatively unchanged (OR: 1.4, 95%CI: 0.6 to 3.3). At the first review visit, the practice of KMC at night and outside their homes was done by fewer mothers than those who had indicated they would on discharge (93.1% vrs 87.9% and 78.1% vrs 58.1 respectively). However, these proportions significantly got higher with each visit with odds of 1.7 (95%CI: 1.2 to 2.6, p = 0.005) and 2.4 (95%CI: 1.7 to 3.3, p < 0.001) after adjusting for weight.

**Table 4:** KMC practice and attitudes at follow-up visits

	Follow-up visit n(%)				Crude analysis		Weight adjusted analysis	
	
	First (n = 182)	Second (n = 175)	Third (n = 170)	Fourth (n = 157)	OR (95% CI)	p-value	OR (95% CI)	p-value
Mother practicing KMC	181 (99.5)	172 (98.3)	165 (97.1)	148 (94.3)	0.4 (0.1-0.8)	0.009	1.4 (0.6-3.3)	0.433
Practicing KMC at night	160 (87.9)	162 (92.6)	163 (95.9)	144 (91.7)	1.2 (0.9-1.6)	0.113	1.7 (1.2-2.6)	0.005
Practicing KMC outside Home	106 (58.2)	126 (72.0)	142 (83.5)	142 (90.4)	3.0 (2.2-3.9)	< 0.001	2.4 (1.7-3.3)	< 0.001
Spouse knowing about KMC?	157 (82.5)	151 (86.3)	145 (85.3)	146 (93.0)	1.3 (1.0-1.7)	0.063	1.0 (0.7-1.4)	0.920
Spouse/Helper practicing KMC?	121 (66.5)	130 (74.3)	128 (75.3)	130 (82.8)	1.7 (1.4-2.2)	< 0.001	1.6 (1.1-2.4)	0.007
Cooking preventing KMC	30 (16.5)	30 (17.1)	26 (15.3)	24 (15.3)	0.8 (0.6-1.1)	0.251	0.8 (0.5-1.2)	0.282
Strange looks preventing KMC	7 (3.8)	5 (2.9)	8 (4.7)	3 (1.9)	0.9 (0.6-1.3)	0.543	1.0 (0.6-1.7)	0.934
Baby suckling	172 (94.5)	168 (96.0)	163 (95.9)	152 (96.8)	1.4 (0.8-2.6)	0.224	1.1 (0.5-2.4)	0.707
Baby taking EBM	150 (82.4)	145 (82.9)	147 (86.5)	129 (82.2)	1.0 (0.8-1.3)	0.875	1.6 (1.1-2.3)	0.011
Baby taking water	6 (3.3)	2 (1.1)	2 (1.2)	1 (0.6)	0.1 (0.0-0.8)	0.031	0.0 (0.0-0.6)	0.023
Baby taking other feeds	7 (3.8)	6 (3.4)	7 (4.1)	5 (3.2)	1.1 (0.3-4.0)	0.931	1.0 (0.4-2.8)	0.944
Weight (kgs) mean(sd)	1.6 (0.3)	1.8 (0.4)	2.0 (0.4)	2.3 (0.5)	_	< 0.001	_	_

The proportion of spouses who knew about KMC did not increase between discharge (82.2%) and first visit (82.5%). This proportion only increased insignificantly over the follow up visits (OR: 1.3, 95%CI: 1.0 to 1.7, p = 0.063). However, the proportion of mothers who had either their spouses or helpers assisting them with KMC increased significantly from 66.5% to 82.8% over the duration of the four visits (OR: 1.6, 95%CI: 1.1 to 2.4, p = 0.007).

A very high proportion of the mothers suckled their infants at the first review visit and this proportion did not change significantly over the four visits (p = 0.707). Mothers were more likely to add expressed breast milk to their babies' feeding over the four visits. Though very few gave added water to their infants this proportion significantly decreased over the four visits from 3.3% to 0.3% (p = 0.023).

The birth weight and discharge weights of the neonates were not significantly different (p = 0.142). Over the four week period from discharge to the end of the reviews the neonates gained 23.7 g per day (95%CI: 22.6 g to 24.7 g per day).

## Discussion

Attitudes, practices and perceptions of KMC in this study showed significant improvement from admission through discharge and follow up visits among mothers. KMC has been found to promote breastfeeding in several studies,[7,15,16] and in this study breastfeeding was continued and sustained among most mothers. Ninety four percent of mothers were exclusively breastfeeding on discharge and they continued throughout the follow up period. These findings are consistent with a study in a tertiary care hospital in Brazil [7] where 108 (88%) LBW infants were exclusively breastfeeding on discharge with 87% still breastfeeding at 1 month. Whereas giving water and other feeds are discouraged during breastfeeding, the few mothers who continued to do so at discharge subsequently discontinued the practice on follow-up visits and counselling.

As mothers reported for follow up visits breastfeeding counselling was reinforced and mothers were more likely to change their wrong attitudes and practices. KMC has very high exclusive breastfeeding rates and where exclusive breastfeeding is uncommon among LBW infants, KMC may bring about an increase in breastfeeding prevalence and duration, with consequent benefits for growth and survival [8,15,17].

Perhaps not very surprising given the recent start of KMC in Ghana, very few mothers knew about KMC at recruitment. This notwithstanding, majority of them practiced KMC after the nurse had explained it to them. Two relatively young primiparous women were however still not comfortable with handling their tiny infants and as such declined to practice KMC. The mothers practicing KMC were also willing to recommend it to other mothers. These findings are consistent with results from a study in rural Ghana [18] involving 635 women from six districts. In that study, most of the women easily understood the KMC concept when they saw a picture of another mother practicing KMC and were willing to try it if it was good for the baby. In our study over 90% of mothers attested to the fact that KMC had been beneficial to them.

KMC practice outside the home was not acceptable to a relatively large proportion of mothers at discharge. The usual practice of carrying newborns in Ghana is for mothers to wrap them against their backs and not on their chest. In this study, many mothers at recruitment felt KMC will not be acceptable in the community because of this difference. However, KMC practice remained relatively stable with each visit. Mothers reported becoming more comfortable with the practice of KMC outside their homes and the proportion of helpers and spouses supporting them with KMC increased significantly with each follow up visit. The increased uptake of KMC could be linked to the fact that, the mothers as they said themselves, explained KMC to anyone in the community who asked about it. The community may have accepted the reasons given by the mothers, thereby indirectly reinforcing the mothers' resolve to continue KMC practice. These results are consistent with a study by Ruiz-Pelaez et al in 2004, which revealed that KMC produced a parental sense of fulfilment and improved confidence of mothers and caregivers as they were empowered by KMC to care for their preterm or low birth weight babies [5]. Cattaneo et al [19] in three different tertiary hospitals in Ethiopia, Indonesia and Mexico, said KMC at all three facilities was considered feasible and mothers expressed a clear preference for KMC. This confirmed that in-hospital KMC for low birth weight babies was feasible in different settings, and acceptable to mothers of different cultures.

The mean weight gain of 23.7 g per day during follow up in this study was comparable to the study by Cattaneo et al [19] (21.7 g per day) but higher than that of Lima et al [7] in Brazil (15 g per day). The weight gain was lower in the study by Lima et al probably because it included averaged weight gain while on admission, the period for which neonates in our study experienced relatively no weight gain.

A major strength of our study is the low dropout rate of less than 20% (including one death) compared to what was expected over the four weeks of follow up. However, in spite of these significant findings our study has notable limitations. First, the mothers and babies lost to follow up could have biased the results, especially if mothers who discontinued KMC at home may have decided not to attend the follow up visits. Secondly, the mothers and caregivers were interviewed by healthcare personnel in a hospital setting. This may have influenced their responses at follow up visits. Also generalisability of the study is limited by the extra effort in the form of phone calls made to follow up mothers following discharge from hospital, which may have helped to maintain KMC. Finally, though the mothers were encouraged to practice continuous KMC because of its proven additional benefit compared to the intermittent, many of them chose to do the latter or combine the two. This could have negatively affected the established benefit of KMC to their infants.

## Conclusion

Knowledge of KMC was low among mothers with LBW infants in Kumasi. After introduction to KMC in the Mother-Baby Unit, a very high proportion of mothers continued practicing at home. The dropout rate was significantly low (compared to expected dropout rate) at less than 20% over the four weeks of follow up. There was high spousal support in the practice of KMC at home. Community attitude did not seem to affect the mothers' practice of KMC at home. Mothers rather grew progressively bolder to perform KMC outside their homes with time. Babies gained optimal weight and maintained a high proportion of exclusive breastfeeding whiles their mothers practiced KMC at home unsupervised. We recommend wider public health campaigns to sensitise the public about the importance of KMC and improve acceptability of the practice.

The positive benefits of KMC are increasingly well-known and these findings highlight the need to address issues of overcrowding at tertiary centres by strengthening peripheral sites to manage these small babies and provide appropriate follow up services for mothers and their babies. More space for KMC would mean fewer deaths, more rapid discharge home and also reduce nursing workload.

## Competing interests

The authors declare that they have no competing interests.

## Authors' contributions

SBN and PNLW conceived, planned, carried out the study and co-headed the drafting of the manuscript. KK conceived, planned and contributed to drafting the manuscript. RO and AY carried out the study and contributed in drafting of the manuscript. SBN carried out the statistical interpretation. JL and GPR supervised the study and contributed to drafting the manuscript. All authors have read and approved the final manuscript.

## Pre-publication history

The pre-publication history for this paper can be accessed here:

http://www.biomedcentral.com/1471-2393/11/99/prepub
